# Understanding hepcidin for iron management in pregnancy

**DOI:** 10.1111/tme.13125

**Published:** 2025-01-28

**Authors:** Sarah Rosson, Sue Pavord

**Affiliations:** ^1^ SUWON (Surgery, Oncology and Womens), Department of Haematology University of Oxford Oxford UK

**Keywords:** anaemia, hepcidin, iron deficiency anaemia, iron therapy, iron deficiency, pregnancy

## Abstract

Iron deficiency anaemia (IDA) poses a significant health challenge during pregnancy, affecting up to 30% of pregnant women in the UK. It has been linked to poor health outcomes for the mother, foetus, and the infant. Despite its prevalence and impact, current diagnostic and therapeutic approaches are limited. Ensuring an adequate iron status in pregnancy requires prompt investigation and treatment whilst avoiding excessive iron supplementation and its associated side effects. Hepcidin, a key regulator of iron trafficking in the body, has emerged as a promising candidate for tailoring iron supplementation to individual needs and responsiveness. However, current research on hepcidin‐based approaches yields mixed findings, necessitating a comprehensive review to elucidate its potential utility in guiding iron therapy for pregnant women with IDA. This literature review seeks to synthesise existing evidence to explore the role of hepcidin in personalised iron supplementation for pregnant women with IDA and to identify avenues for future research to pave the way for improved management of IDA in pregnancy.

**What is known about this topic?**

- Iron deficiency anaemia (IDA) affects up to 30% of pregnant women in the UK and poses a significant risk to both mothers and babies.
- Pregnancy increases iron demand, often exceeding dietary intake and maternal iron stores, leading to IDA in the absence of supplementation.
- Hepcidin, a key regulator of iron trafficking, negatively regulates iron absorption.
- Iron supplementation is essential to restore iron stores and haemoglobin levels during pregnancy.
- Current treatment options include:
  - Oral iron supplements, which is limited by sub‐optimal absorption and compliance issues.
  - Parenteral therapy, which is effective but comes with high costs, logistical challenges and unknown long‐term effects.

**What is new?**

- Recent research on using hepcidin to guide iron supplementation in pregnancy has yielded mixed results, highlighting the need for a comprehensive review.
- Hepcidin may be helpful in indicating responsiveness to iron therapy, as raised hepcidin reduces capacity for absorption.
- Studies have shown that hepcidin levels remain elevated for up to two days following oral iron and two weeks following intravenous iron, suggesting that oral iron supplements should be suspended during this period as absorption will be poor.
- Excess iron supplementation may be harmful, resulting in oxidative stress. Optimising iron therapy is a balance between maximising benefits while minimising adverse effects.

**What are the key questions for future work on the topic?**

- Can serum hepcidin be used to guide treatment of iron deficiency in pregnancy?
- How can we standardise hepcidin measurement techniques and establish reference ranges specific to pregnancy?
- Can urinary hepcidin effectively serve as a non‐invasive biomarker for assessing iron status and treatment responsiveness in pregnant women?
- What are the optimal hepcidin thresholds for guiding iron therapy in pregnant women, and how can these be integrated into clinical practice?
- How can early detection and prevention strategies for IDA in pregnancy be improved to mitigate adverse maternal and fetal outcomes?
- Does adjusting dose and frequency of iron supplementation based on hepcidin levels improve efficacy and reduce side effects, compared to standard iron supplementation?

## 1 INTRODUCTION

Iron is the fourth most abundant element in the Earth's crust. It is critical to human biology and has been used therapeutically for millennia. However, its significance to medicine extends beyond its utility; iron deficiency (ID) remains a global health concern, affecting more than 1.2 billion people worldwide.^1^ Within the pregnant population, iron deficiency anaemia (IDA) imposes a considerable burden, affecting between 25% and 30% of pregnant women in the UK alone.^2^ It is the foremost cause of anaemia in pregnancy^3^ and has been linked to poor health outcomes in the mother (such as postnatal depression^4^ and postpartum haemorrhage^5^), as well as the foetus (low birth weight and pre‐term birth)^6^ and the infant (impaired neurological development).^7^

Current treatments include oral and parenteral iron supplementation; both have limitations. Systemic absorption and adherence to oral supplementation are sub‐optimal, and parenteral therapy is costly for the health service and not without risk to the patient.^8^ Furthermore, excess iron supplementation may also be associated with adverse maternal^9^ and fetal outcomes,^10^ reinforcing the need to individualise iron therapy.

A biomarker that has been proposed to guide iron requirements is hepcidin. Hepcidin, a portmanteau of hepatic bactericidal protein,^11^ has emerged as a promising candidate for guiding iron supplementation in various clinical settings, including pregnancy. It is a peptide hormone that controls iron trafficking in the body by regulating the efflux of iron into plasma.^12^, ^13^ Hepcidin degrades the iron transporter, ferroportin, decreasing iron plasma concentrations and systemic iron availability. It has been proposed that the hepcidin‐ferroportin axis could be used to guide iron therapy to ensure that oral iron supplementation (and its associated side effects) would only be administered when it would have therapeutic benefits.

This review explores the existing evidence on hepcidin's utility as a biomarker for directing iron supplementation in pregnant women with IDA, based on individual needs and physiological status.

## 2 BACKGROUND

In the body, iron requirements are met through a combination of dietary uptake and iron recycling mechanisms. Iron is lost through menstruation and other bleeding but there is no physiological mechanism for iron excretion. Dietary iron is present in two primary forms: heme iron, predominantly found in animal products, and non‐heme iron, prevalent in plant‐based foods. Upon reaching the duodenum, ferric iron (Fe^3+^) undergoes reduction to ferrous iron (Fe^2+^) at the apical membrane^14^ of duodenal enterocytes. This reduction process, facilitated by duodenal cytochrome b, enables iron to enter the cell via the divalent metal transporter (DMT‐1).^14^

Once inside the enterocyte, iron is either stored as ferritin, the principal iron storage protein, or exported to the blood through ferroportin, the only known iron exporter in humans.^14^ In the circulation, iron binds to transferrin, the main protein transporter in the blood, for delivery to various tissues, including the bone marrow for erythropoiesis.^14^

Hepcidin induces the degradation of ferroportin^14^ to negatively regulate iron absorption, particularly in the duodenum. As such, elevated hepcidin levels are associated with decreased iron absorption, thereby influencing systemic iron status.

During pregnancy, the total iron requirement is over 1000 mg.^15^ This demand often exceeds the capacity of both maternal iron stores and dietary iron absorption. Consequently, insufficient iron availability can lead to progressive iron depletion, followed by cellular iron deficiency, impaired erythropoiesis and eventually IDA.

Anaemia arises when there is insufficient haemoglobin (Hb) for the blood to adequately oxygenate organs and tissues, as there is a reduced oxygen‐carrying capacity. According to the WHO, anaemia is a Hb concentration in the 5th percentile.^16^ However, determining anaemia during pregnancy poses challenges due to variations in normal Hb values. Current guidelines define anaemia in pregnancy as Hb levels below 110 gL^−1^ in the first and third trimesters and below 105 gL^−1^ in the second trimester.^16^

### 2.1 Challenges in diagnosing IDA in pregnancy

Diagnosing IDA in pregnancy is inherently challenging due to the non‐specific nature of symptoms and limitations of diagnostic markers. Iron deficiency anaemia in pregnancy often manifests with symptoms, such as fatigue, pallor, weakness, headache, palpitations, dizziness, dyspnoea, and irritability.^17^ Although some individuals with IDA may exhibit characteristic unusual cravings like pagophagia (a pathologic craving for ice) and other forms of pica, such as craving dirt,^17^ most of the symptoms are not specific to IDA and can overlap with common pregnancy‐related complaints.

Definitive diagnosis of IDA relies on the assessment of Hb levels and serum ferritin concentrations. However, interpreting ferritin levels is challenging due to pregnancy‐related changes in inflammatory markers, which can significantly influence ferritin concentrations.^17^ Normal serum ferritin levels in pregnancy may not accurately reflect iron status due to the rise in acute phase proteins, including ferritin, in response to inflammation.^18^ Moreover, the lack of consensus on pregnancy‐specific ferritin thresholds for diagnosing ID^19^, ^20^ contributes to variability in diagnostic criteria across guidelines.^21^ This poses a risk of under‐diagnosis, meaning that ID may not be picked up until it affects Hb production and becomes IDA.

Delaying the diagnosis of ID until it progresses to anaemia may have adverse neurodevelopmental consequences for the baby. Research suggests that ID in early pregnancy can result in persistent neurodevelopmental alterations despite iron repletion.^22^ Thus, early identification and management of ID before it escalates to IDA are crucial for mitigating these risks.

Current guidance is that all women should have a full blood count taken at the first antenatal (booking) appointment and 28 weeks gestation.^23^ Oral iron therapy is proposed as both a therapeutic measure and a diagnostic tool for those with anaemia. Within 2 weeks of treatment, there should be a rise in Hb to confirm IDA. If no improvement in Hb is seen, IDA is not ruled out, but further investigation is needed, involving serum ferritin and folate in the first instance.^17^

Alternative markers to indicate iron status in early pregnancy are under investigation. Hepcidin has greater sensitivity than Hb for detecting ID in pregnancy, correlating well with iron status indicators like ferritin and soluble transferrin receptor.^24^ Percent transferrin saturation (TSAT) has been proposed as a potential indicator of iron deficiency,^25^ but its utility is limited by various factors, including diurnal variation, infection, and inflammation.^26^ Further research is needed to establish validated reference thresholds and standardised assays for using hepcidin and TSAT as reliable markers for early detection and management of ID in pregnancy.

### 2.2 Challenges and limitations of the current approach to iron supplementation during pregnancy

Concerns have been raised about the potential harm of iron supplementation. Iron metabolism is linked to oxidative stress^27^ and increased ferritin is associated with higher risk of gestational diabetes^28^ and exacerbation of existing infections. In low‐income regions and areas where malaria is endemic, iron supplementation can heighten the risk of gastrointestinal infections^29^ and malaria.^30^ Iron deficiency is recognised as a greater risk than iron excess during pregnancy,^31^ and although over‐supplementation is unlikely to reach levels that would cause harm, the need to rapidly increase Hb should be counterbalanced by exposure to free iron.

Oral iron is the preferred route to restore Hb and iron stores in pregnancy, due to its ease of availability and low cost. However, oral iron salts frequently cause gastric irritation, leading to nausea and epigastric discomfort that is dose‐limiting in up to a third of patients.^32^ Compliance and intolerance issues pose significant barriers to its efficacy although recommended alternate‐day regimes improve absorption and reduce side effects.^17^, ^33^ This is further supported by observed changes in serum hepcidin, which remain elevated at 24 h, but not at 48 h, following 60 mg elemental iron.^34^ Studies are underway to assess the transferability of this data to pregnancy.

Parenteral iron therapy offers a faster rise in Hb and ferritin levels than oral iron, and receivers are less likely to experience adverse events.^35^ However, it is not licenced for use in the first trimester due to potential risks of toxicity to developing structures and the financial burden of parenteral therapy, including nursing time, supplies, and patient inconvenience, limits its widespread adoption as a primary treatment modality.^15^

Current WHO guidance recommends weekly oral iron in non‐anaemic pregnant women if daily iron is not acceptable due to side effects.^36^ This is based on the debated “mucosal block” theory: while hepcidin governs iron regulation at a systemic level, the mucosal block is postulated to operate cellularly within the gut, suggesting that when enterocytes are exposed to substantial amounts of iron, they absorb and store some of it.^37^ Once saturated, these cells limit further absorption until they are replaced by new, iron‐depleted enterocytes, which naturally takes 5 to 6 days.

Given that serum hepcidin subsides after 48 h, any sustained absorption inhibition would be consistent with the “mucosal block” theory. However, recent research in non‐pregnant populations has challenged this idea, finding no evidence of reduced iron absorption 48 h after a prior dose.^33^ This should be assessed in pregnant populations to determine if women experiencing adverse effects from iron supplementation can receive a more effective regimen than the WHO recommends.

### 2.3 Hepcidin as a biomarker in non‐pregnant populations

Hepcidin varies in response to iron status, erythropoiesis, hypoxia, and inflammation.^38^, ^39^, ^40^ Knowledge of circadian fluctuations in hepcidin is already being leveraged to optimise absorption in oral iron therapy, favouring morning dosing when hepcidin is lowest.^17^, ^41^

Hepcidin has already been proposed as a biomarker to guide iron therapy for non‐pregnant populations in specific clinical scenarios, including cases of haemodialysis patients,^42^ chronic rheumatologic disease,^43^ and inflammatory bowel disease (IBD).^8^ Aligning supplementation with an individual's capacity to absorb iron could improve treatment efficacy, avoid adverse effects of oral iron therapy in individuals with elevated hepcidin levels,^8^ and prevent unnecessary admissions and workday losses associated with parenteral iron therapy.

A recent small study observed that hepcidin remained elevated for up to 2 weeks post‐infusion,^44^ suggesting that outcomes may be improved by interrupting oral iron supplements during this period when absorption will be poor and gastrointestinal side effects heightened from excess iron presence in the gut.

A study on haemodialysis patients found that oral iron therapy was efficiently absorbed in individuals with normal hepcidin levels, suggesting that integrating hepcidin measurements into treatment plans could enhance anaemia management in this population.^42^ Similarly, in patients with chronic rheumatologic disease, baseline hepcidin levels served as a predictor of Hb response to iron therapy, highlighting the potential for personalised treatment approaches based on hepcidin levels.^43^ Another study looking at patients with active IBD undergoing anti‐inflammatory treatment is attempting to determine if tailoring treatment based on hepcidin levels leads to better outcomes; the results have not yet been published.^8^ However, the extent to which this is relevant to pregnant populations is up for debate, as harms posed by IDA in pregnancy may be comparatively more significant than the risk of excess iron exposure, and there are suggestions of a “fetal factor” that may contribute to hepcidin suppression during pregnancy.^24^

Despite its potential utility, several challenges and limitations are associated with measuring hepcidin levels. Different assays have generated discrepancies,^45^ reference ranges are limited even in non‐pregnant populations, and those derived from healthy pregnant populations in high‐resource settings may not be universally applicable.^46^ As such, the high cost, apparent diurnal variation,^47^ and the need for standardisation across measured values^48^ pose significant barriers to hepcidin's use as a diagnostic tool.

In summary, while hepcidin holds promise as a biomarker to guide iron therapy across various clinical contexts, these studies may not directly translate to pregnancy scenarios since the urgent need to rapidly increase Hb levels in pregnant patients is believed to outweigh the harm associated with excess iron. Moreover, addressing the challenges and limitations associated with its measurement is essential for effective integration into clinical practice. Further research and standardisation efforts are warranted to realise the full potential of hepcidin‐guided iron therapy in optimising patient care.

### 2.4 Hepcidin during pregnancy

Pregnancy imposes a significant increase in iron requirements to support placental and fetal growth, and the rise in RBC mass in line with plasma expansion.^49^ In normal pregnancy, hepcidin levels are lowered to accommodate the heightened demand for iron.^50^ Failure to fulfil this increased iron demand results in the depletion of maternal iron stores,^51^, ^52^, ^53^ leading to IDA and potentially exacerbating adverse outcomes for both mother and fetus. Besides its role in blocking intestinal iron uptake, hepcidin is also believed to inhibit the placental transport of iron.^13^, ^38^

Hepcidin concentrations decline across pregnancy,^24^ reflecting the shifting iron demands. In the first trimester, iron requirements are relatively low due to the recent cessation of menstruation,^54^ and serum hepcidin concentrations resemble those of non‐pregnant women.^55^, ^56^, ^57^ However, as pregnancy progresses, hepcidin concentrations decline to facilitate enhanced iron absorption and delivery to support fetal growth.^58^ Towards the end of the third trimester, hepcidin levels remain low to undetectable, ensuring maximum iron availability for maternal and fetal needs.^56^, ^57^ A recent longitudinal cohort study discovered that maternal ID does not fully account for the observed reduction in hepcidin levels during pregnancy, suggesting that an as‐yet unidentified “fetal factor” may contribute to the suppressed maternal hepcidin expression.^24^

A few studies have examined hepcidin concentrations in pregnant women with IDA. One such study reveals multi‐directional changes across pregnancy subgroups.^59^ While some women exhibit raised hepcidin levels alongside lowered erythropoietin (EPO) concentrations, others display low hepcidin levels with increased EPO concentrations. This heterogeneity underscores the complexity of IDA in pregnancy and suggests varying responsiveness to oral iron therapy. Further research should be performed to assess the correlation of hepcidin levels with response to oral iron therapy.

Several studies have assessed the hepcidin level in pregnant women with and without anaemia,^24^, ^44^ finding that serum hepcidin levels are low in physiological and ID pregnancies.^24^, ^44^ In the case of anaemia, suppressed hepcidin can be used with other iron status indicators, such as TSAT and ferritin to differentiate between IDA and different types of anaemia, where hepcidin may not be as low. Thus, indicating that women with anaemia and low hepcidin are likely to have IDA, and respond to oral iron supplementation. Achieving concordance and standardisation in hepcidin reference ranges and measurement approaches is paramount to facilitating the clinical utility of hepcidin as a biomarker for iron therapy in pregnant women with IDA.

### 2.5 Hepcidin as a biomarker in pregnant populations

A few studies have investigated the feasibility of using hepcidin levels to guide iron therapy in pregnant women. Bah and colleagues used a “screen‐and‐treat” approach, where oral iron supplementation was administered based on serum hepcidin levels.^60^ Oral iron supplementation was given to women with serum hepcidin <2.5 μgL^−1^, that is, those where serum hepcidin is suppressed such that iron can effectively be absorbed. The control group received the WHO's recommended amount of 60 mg of iron daily, while two “screen‐and‐treat” groups received 60 mg and 30 mg daily. The amount of Hb at day 84 of treatment was compared. The “screen‐and‐treat” value was determined based on the WHO's recommendation of 30–60 mg of elemental iron, where 60 mg should be used in settings where anaemia in pregnancy has a prevalence of 40% or higher.^61^

However, contrary to expectations, this approach appeared less effective than the standard World Health Organisation (WHO) guidelines for treating IDA in pregnant women. The trial was still upheld as a non‐inferiority trial, as the primary outcome of Hb assessment at day 84 was within the non‐inferiority margin, however, the prevalence of IDA at day 84 was higher in the intervention group, indicating that the intervention was less effective at treating IDA. More concerning however, the study also brings the limited efficacy of the recommended oral iron treatment for IDA in pregnancy into harsh perspective, as the prevalence of anaemia at day 84 in the reference group was still 45%.^60^

Several factors may contribute to the discrepancies in hepcidin‐based iron therapy. Weekly screening may prove inefficient for capturing short‐term hepcidin dynamics, and the lack of standardised hepcidin thresholds warrants re‐evaluation to distinguish iron absorbers from blockers. Bah and colleagues suggested that bolus doses of the oral iron supplement could override physiological mechanisms of hepcidin‐induced iron blockade, hence iron continued to be absorbed even when hepcidin was high.

Although data from this trial do not support hepcidin‐based treatment, further studies to refine hepcidin‐based iron therapy approaches are warranted, as understanding of hepcidin and optimal iron supplementation evolves. It is suggested that a higher hepcidin threshold should be used to identify the appropriate iron dosage, as there is a wealth of benefits of oral iron supplementation to maternal and fetal health and limited evidence of harm from current supplementation doses. Thus, withholding iron supplementation based on hepcidin concentrations that are low but still above an identified threshold may not provide overall benefits, despite a slight reduction in adverse symptoms. This should be confirmed in follow‐up studies, along with increasing screening frequency, reassessing hepcidin thresholds, and exploring alternative forms of iron administration.

### 2.6 Future studies

There is an acute need for increased focus on the prevention of IDA in pregnancy, considering its significant implications for maternal and fetal health. Future research should define hepcidin, serum ferritin and TSAT thresholds indicative of IDA in pregnancy and assess the impact of integrating these into treatment algorithms. Emphasis should also be placed on identifying predictive markers and early detection methods for ID in pregnancy to mitigate the maternal and fetal consequences of iron depletion. Additionally, it would be beneficial to determine if hepcidin levels can accurately reflect a pregnant individual's iron absorption capacity.

Further research should investigate the potential existence of a “fetal factor” that may influence hepcidin suppression and explore whether local gut inflammation may limit iron absorption. The effectiveness of alternate‐day dosing, already shown to have improved outcomes in non‐pregnant individuals, should be studied for its transferability to ID and IDA in pregnancy.

Other potentially promising areas for research include assessing urinary hepcidin as a potential non‐invasive biomarker. Hepcidin is excreted in urine^62^ and has been observed to have a linear relationship to iron stores over a wide range of serum ferritin concentrations,^54^ making it a potential tool for monitoring iron status and treatment response. Furthermore, clinical trials exploring novel hepcidin antagonists^48^ offer potential therapeutic opportunities that warrant further exploration for their efficacy and safety in managing IDA during pregnancy.

Addressing these research gaps will enhance our ability to effectively prevent, diagnose, and treat IDA in pregnant women.

## 3 CONCLUSION

In conclusion, this article serves as a thought piece, reviewing the literature that explores the role of hepcidin in improving the treatment of IDA in pregnancy. Our review highlights that IDA in pregnancy remains a significant global health concern, with significant implications for maternal and fetal well‐being. Despite advancements in diagnostic and therapeutic approaches, challenges persist in accurately identifying and effectively treating IDA during pregnancy.

Although hepcidin is a promising candidate for optimising iron therapy during pregnancy, current research into hepcidin‐based approaches for managing IDA in pregnancy has yielded mixed results. Some studies demonstrated promise, yet others reveal limitations in hepcidin‐based treatment strategies, emphasising the need for standardisation of measurement techniques and reference ranges. Notably, many of the studies considered are small in scale and carry several limitations, underscoring the need for further investigation to refine hepcidin‐based treatment strategies in pregnant populations.

Finally, there is a pressing need to prioritise prevention strategies for IDA in pregnancy. By addressing these research gaps and challenges, we can enhance our ability to prevent, diagnose, and treat IDA in pregnant women, ultimately improving maternal and fetal outcomes.

## AUTHOR CONTRIBUTIONS

All authors contributed to the development of this work. Sarah Rosson prepared the first draft of the manuscript, which was reviewed and commented on by all authors. All authors participated in revising the manuscript and approved the final version for submission.

## CONFLICT OF INTEREST STATEMENT

Unrelated to this manuscript, Sue Pavord has received educational fees and sponsorship from Pharmacosmos and Vifor Pharma for talks and advisory boards.
